# Multisyringe Flow Injection Analysis of Tropomyosin Allergens in Shellfish Samples

**DOI:** 10.3390/molecules26195809

**Published:** 2021-09-25

**Authors:** Bruno Coulomb, Fabien Robert-Peillard, Najib Ben Ali Gam, Salwa Sadok, Jean-Luc Boudenne

**Affiliations:** 1 Aix Marseille Univ, CNRS, LCE, Marseille, France; bruno.coulomb@univ-amu.fr (B.C.); fabien.robert-peillard@univ-amu.fr (F.R.-P.); benaligamnajib@yahoo.fr (N.B.A.G.); 2 Laboratoire de Biodiversité et Biotechnologies Marines, Institut National des Sciences et Technologie de la Mer, Centre La Goulette, Port de Pêche, Tunis 2060, Tunisia; salwa.sadok@instm.tn

**Keywords:** tropomyosin, seafood, glutamic acid, MSFIA, fluorescence

## Abstract

This paper presents the development and the application of a multisyringe flow injection analysis system for the fluorimetric determination of the major heat-stable known allergen in shrimp, rPen a 1 (tropomyosin). This muscle protein, made up of 284 amino acids, is the main allergen in crustaceans and can be hydrolyzed by microwave in hydrochloric acid medium to produce glutamic acid, the major amino acid in the protein. Glutamic acid can then be quantified specifically by thermal conversion into pyroglutamic acid followed by chemical derivatization of the pyroglutamic acid formed by an analytical protocol based on an OPA-NAC reagent. Pyroglutamic acid can thus be quantified between 1 and 100 µM in less than 15 min with a detection limit of 1.3 µM. The method has been validated by measurements on real samples demonstrating that the response increases with the increase in the tropomyosin content or with the increase in the mass of the shrimp sample.

## 1. Introduction

High consumption of seafood has often been associated with health benefits by providing protection and/or symptom-relieving effects for cardiovascular disease [1], colorectal cancer [2], and inflammatory diseases such as rheumatoid arthritis and inflammatory bowel diseases [3,4], psychological effects [5], and has also been associated with beneficial outcomes in relation to infant development, mental health, arthritis, and cognitive decline [6,7]. Seafood is indeed a source of essential nutrients, particularly high-quality proteins, retinol, vitamin D, vitamin E, iodine, selenium, and omega-3 long-chain polyunsaturated fatty acids (LC-PUFAs), such as ecosapentaenoic acid (EPA) and docosahexaenoic acid (DHA), which assists in the favorable evolution of many diseases [8]. However, seafood may also be contaminated with components present in the aquatic environment such as micro-organisms, algae biotoxins, and chemicals (for example, methyl mercury, dioxins, and polychlorinated biphenyls) [9,10]. 

In addition, seafood can cause food allergy, which today affects 2.5% of the general population around the world [11,12]. The most reported signs are generalized itching, development of urticaria, and swelling of the lips and tongue, but seafood allergy may also lead to pulmonary and/or gastrointestinal symptoms, and anaphylactic shocks [13,14,15]. The major seafood allergens are parvalbumin and tropomyosin in fin fish and shellfish, respectively, representing the two largest classes of animal-derived allergens according to the database of allergen families AllFam [16,17,18]. The main crustaceans involved in food allergies are shrimp, crab, and lobster. For example, shellfish allergy affects 2% of children and 3% of adults with food allergy in France [19], and in Tunisia 12.7% of children are affected by food allergy, including 9.2% with allergy to shellfish and especially shrimp [20]. Among these allergens, tropomyosin, which is a muscle protein with a molecular weight that varies from 34–36 KDa, has been identified as the major allergen in shrimp [21]. Allergic responses induced by tropomyosin occur when this protein enters the intestinal tract and crosses the mucosal membrane surfaces where it is processed by macrophages and comes into contact with T and B lymphocytes. Enhancement of the production of protein-specific immunoglobulin E (IgE) antibodies—responsible for type I hypersensitivity reactions—can result from this reaction. These antibodies may then bind to mast cells or basophil granulocytes and induce the cross-linking of FcεRI, resulting in cell degranulation and release of numerous allergenic mediators (such as histamine). These compounds may then cause changes in cell migration and dilatation of blood vessels and/or muscle contraction, leading to the symptoms observed during an allergic reaction [11,12].

Several bioanalytical methods for the detection of tropomyosin in shellfish have been developed, such as enzyme-linked immunosorbent assay (ELISA) [22] or polymerase chain reaction (PCR) methods [23,24] which have many drawbacks such as indirect detection of allergens, possibility of false positive/negative results, need for intensive and sophisticated experimental steps or even long reaction or sample preparation times. High-performance chromatography coupled with mass spectrometry (HPLC/MS) methods have also been reported and have emerged as a useful tool for allergen screening [25,26], but these analyses require complex and expensive equipment. The development of rapid, sensitive, and low-cost analytical methods for the detection and quantification of tropomyosin has therefore become a necessity.

The amino acid composition of tropomyosin reveals a preponderance of glutamic acid [27]. It is therefore possible to indirectly determine tropomyosin by quantifying glutamic acid. Amino acids are known to respond in different ways when submitted to elevated temperatures, with some able to be broken apart and others able to polymerize or cyclize [28,29]. To limit interference from other amino acids and to ensure specificity of the quantification, glutamic acid can thus be converted to pyroglutamic acid by heat treatment as already shown by several authors [30,31]. Pyroglutamic acid, which has a secondary amine function, can then be quantified by a derivatization procedure using *o*-phthalaldehyde/N-acetyl cysteine (OPA-NAC) reagent after hydrolysis with sodium hypochlorite [32,33]. This procedure can then be automated and integrated into different online analytical systems [34]. Only a few flow systems have been applied to the analysis of organic or inorganic compounds in food samples. Food samples often require complex sample preparation steps; however, the chemical derivatization and detection steps could be easily automated to save lab time and money (less reagent consumption).

In this study, the indirect determination of tropomyosin in shrimp hydrolyzate samples was carried out by an MSFIA system via the sensitive and specific quantification of glutamic acid. The various analytical steps, consisting in conversion of glutamic acid into pyroglutamic acid and then derivatization and quantification by fluorescence of the secondary amine thus formed, were optimized and validated by tests on real samples.

## 2. Results and Discussion

### 2.1. Conversion of Glutamic Acid into Pyroglutamic Acid

**Figure 1:**
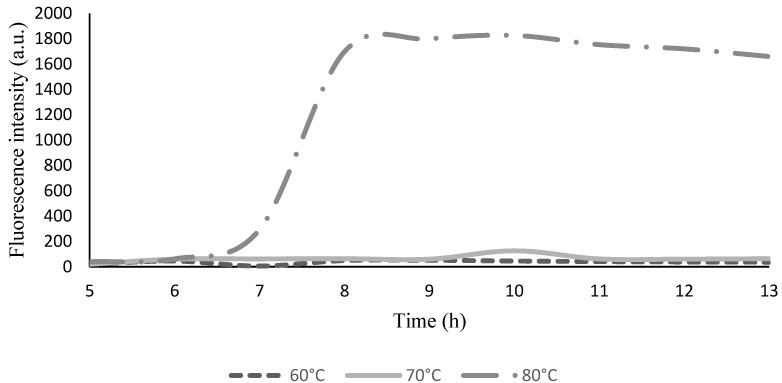
Effect of temperature on pyroglutamic acid (60 µM) formation as a function of time.

The optimum temperature for the conversion of glutamic acid into pyroglutamic acid was determined from a glutamic acid standard, within a temperature range between 60 and 80 °C, and a heating time between 5 and 13 h. The response studied was the fluorescence intensity resulting from the overall analytical procedure carried out with the MSFIA system; the greater the fluorescence, the greater the pyroglutamic acid formed (glutamic acid does not react in the derivatization procedure developed). The results are shown in Figure 1.

The results showed that a temperature of 80 °C was needed to convert glutamic acid into pyroglutamic acid. The conversion rate reached a maximum after 8 h of heating and then remained stable. It should be noted that no temperature above 80 °C was tested to avoid the degradation of the pyroglutamic acid.

Extracts resulting from the thermal conversion of glutamic acid were analyzed using UPLC-ESI-MS and MS/MS to identify the transformation products. These analyses led to the identification of pyroglutamic acid (*m*/*z* 128.035) in negative mode and also to the detection of a minority derivative of pyroglutamic acid (*m*/*z* 240.1502) in positive mode. No trace of glutamic acid was detected, indicating that the conversion of glutamic acid to pyroglutamic acid was complete.

### 2.2. Study of Interferences

**Figure 2:**
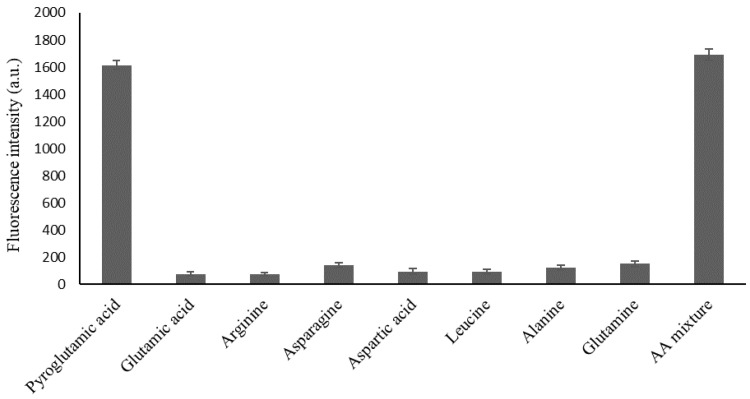
Fluorescence response of various amino acids with the optimized analytical protocol (*n* = 3). Pyroglutamic acid: 50 µM. Amino acids: 50 µM.

The interferences of primary amines were avoided by the addition of an OPA solution at a pH of 7.75 at the beginning of the analytical protocol, as has already been demonstrated in a previous work [33]. The response of the developed MSFIA system was tested on the main amino acids that may occur from the hydrolysis of tropomyosin [27]. The results presented in Figure 2 show that the response of each amino acid tested represented less than 10% of the response of pyroglutamic acid. The fluorescence intensity obtained for pyroglutamic acid alone and that obtained for pyroglutamic acid mixed with amino acids were in agreement with a difference of less than 5%. These results therefore showed that the analytical protocol developed was selective for the determination of pyroglutamic acid.

### 2.3. Analytical Features

The analytical protocol of the MSFIA system was applied on a series of blank solutions and on pyroglutamic acid standards. The method showed a linear fluorescence response up to a concentration of 100 µM with an excellent correlation coefficient (R^2^ = 0.999). The relative standard deviation was determined at 4.66% by measuring a 50 μM pyroglutamic acid standard (*n* = 7 replicates). The limit of detection and the limit of quantification were measured at 1.3 and 4.4 µM, respectively.

The preservation of OPA/NAC reagent has already been studied previously [35]. It had been shown that OPA and NAC solutions were stable for up to four months, provided these solutions were stored separately at 4 °C in the dark.

### 2.4. Validation on Real Samples

#### 2.4.1. Standard Addition of Tropomyosin

**Figure 3:**
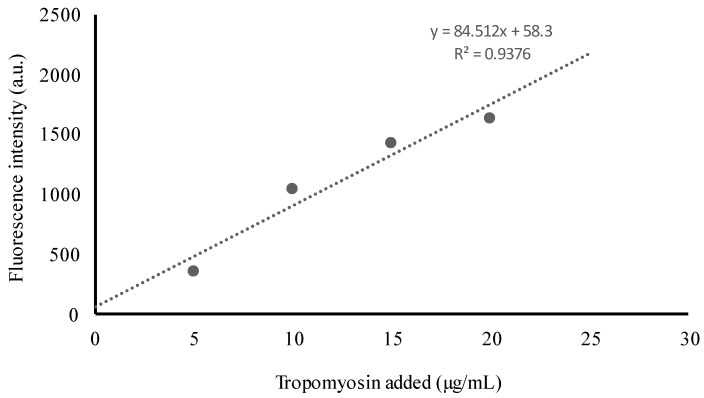
Calibration curve of standard addition of tropomyosin in a real sample.

In order to confirm the reliability and the precision of the method, a shrimp sample, prepared according to the protocol presented in Section 3.2.1, was spiked with increased concentrations of tropomyosin. The results (Figure 3) showed that the fluorescence response increased with the volume of tropomyosin added to the sample with a satisfactory correlation coefficient (R^2^ = 0.938), which reflects that the method is reliable. The results obtained were similar to those obtained by Wang et al. [36] who also conducted standard additions of tropomyosin to shrimp samples.

#### 2.4.2. Validation of the Overall Procedure

**Figure 4:**
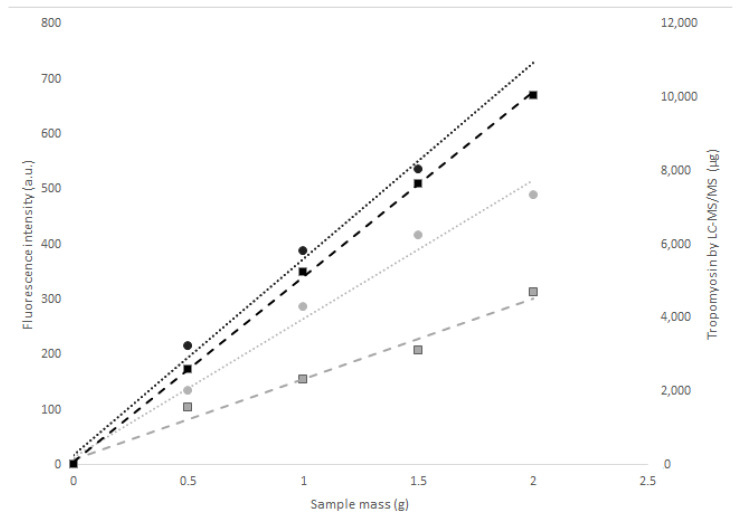
Comparison of calibration curves obtained after application of the MSFIA method based on glutamic acid fluorescence measurements (data represented by circles) and after tropomyosin determination by LC-MS/MS (data represented by squares) on different masses of samples of 2 species of shrimp. In gray: *Peneaus vennamei*; in black: *Melicertus kerathurus*.

Samples of two different shrimp species (*Peneaus vennamei* and *Melicertus kerathurus*) were prepared according to the protocol presented in Section 3.2.1 and hydrolyzed by microwave in 6 M HCl. Glutamic acid was then quantified in the extracts obtained for each species of shrimp, and for different masses of samples, by use of the MSFIA system The results obtained (Figure 4) showed that the fluorescence intensity increased with the mass of the two species studied with very satisfactory correlation coefficients (R^2^ = 0.996 and R^2^ = 0.972 for *Peneaus vennamei* and *Melicertus kerathurus*, respectively).

These two samples were also analyzed according to the procedure described in Section 3.5 (SDS-PAGE profiles presented in Appendix A) and analyzed by LC-MS/MS (as described in Section 3.4). These two methods do not measure the same compounds. Tropomyosin is composed of two polypeptide chains, with five peptides found after digestion [37]. Among them, the peptide with the IVELEEELR sequence has been shown to be the most sensitive and was thus chosen to be a marker of tropomyosin [37]. The alternative method proposed in this paper found glutamic acid to be the most prevalent amino acid in that peptide, and it was thus chosen to be a marker of tropomyosin. Figure 4 shows that, despite the different modes of tropomyosin hydrolysis used between the two methods, a good linearity between both methods was observed, showing that glutamic acid seems to be a good proxy of tropomyosin in seafood. Levels of tropomyosin in *Peneaus vennamei* and *Melicertus kerathurus* as determined by LC-MS/MS were 2335.4 and 5251.4 µg·g^−1^, respectively. Their levels as determined by the alternative method were 2278.8 and 4787.3 µg·g^−1^, respectively, i.e., the difference between the two methods was below 10%.

In view of the simplified proposed method as compared to the time-consuming and expensive current method, this difference is expected to be negligible, and current regulations do not specify a threshold limit. However, more shrimp samples and other seafood samples need to be analyzed according to the same procedures to confirm these promising results.

## 3. Materials and Methods

### 3.1. Reagents

All chemicals used in this study were of analytical grade and used without further purification. Aqueous solutions were prepared with ultrapure deionized water using a Millipore Milli-Q purification system (Millipore, Burlington, MA, USA, resistivity > 18 MΩ cm). Natural shrimp tropomyosin, hydrochloric acid (HCl), sodium citrate and pyroglutamic acid were obtained from Sigma-Aldrich, Saint-Quentin Fallavier, France) and sodium hypochlorite (NaOCl), N-acetylcysteine (NAC) and o-phthaldialdehyde (OPA) were purchased from Acros Organics, IllKirch, France). A 0.05 M borate solution and a 0.2 M phosphate buffer solution were prepared by dissolving sodium tetraborate decahydrate (Na_2_B_4_O_7_·10H_2_O) and di-sodium hydrogen phosphate dodecahydrate (Na_2_HPO_4_·12H_2_O), respectively, in ultrapure water.

OPA-NAC reagent was prepared by dissolving an appropriate amount of OPA in ethanol and then diluting (20:80, *v*/*v*) in a buffered borate NAC solution adjusted to pH = 10.5.

### 3.2. Experimental Protocol

**Scheme 1:**
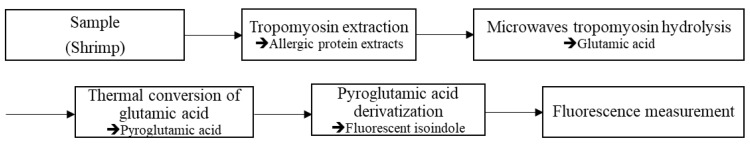
Overall experimental protocol.

The overall experimental protocol for the determination of tropomyosin in shrimps is summarized in Scheme 1.

Briefly, an extraction of the allergenic proteins was carried out by mixing the sample in an aqueous buffer, then the extract containing the tropomyosin was hydrolyzed into glutamic acid with hydrochloric acid in a microwave reactor. The glutamic acid was then thermally converted into pyroglutamic acid able to be quantified by the MSFIA system by derivatization with an OPA-NAC reagent and detection by fluorescence. These different steps are detailed below.

#### 3.2.1. Seafood Sample Preparation

The crustacean species used in this study were purchased from a Tunisian market (shrimp—*Melicertus kerathurus*) and from a French market (shrimp—*Penaeus vennamei*). Allergic protein extracts from crustacean products were prepared according to a previously reported method [36,38], modified as follows: 5 g of muscle were homogenized in 15 mL of a 0.01 M phosphate-buffered saline solution (PBS) containing 3% NaCl for 10 min using an UltraTurax mixer. The homogenate was stirred for 3 h at 4 °C, followed by centrifugation at 12,000 rpm for 20 min. The supernatant was filtered through a 0.45 µm polyethersulfone (PES) membrane and stored at −80 °C prior to analysis.

#### 3.2.2. Hydrolysis of Tropomyosin

In this study, hydrolysis was carried out using a Milestone Start-D microwave (Milestone, Italy) according to the method previously reported by Kabaha et al. [39]. Digestion was carried out using HCl 6 M at 130 °C for 2 h. After hydrolysis, 1 mL of the extract was neutralized with 0.55 mL of NaOH 32% (*w*/*v*). Then, 5 mL of sodium citrate (200 mM) were added and the mixture was filtered through a 0.22 µm membrane filter before analysis.

#### 3.2.3. Conversion of Glutamic Acid

The conversion of the primary amino acid (glutamic acid) into a secondary amino acid (pyroglutamic acid) was carried out according to the method reported by Povoledo and Vallentyne [28], modified as follows: the hydrolyzate obtained previously was heated for 8 h in Pyrex glass tubes at 80 °C in a water bath.

#### 3.2.4. Derivatization Procedure of Pyroglutamic Acid

**Scheme 2:**
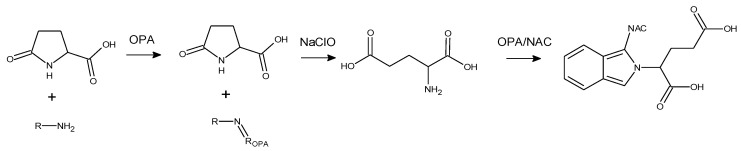
Derivatization of pyroglutamic acid by OPA-NAC reagent.

The derivatization procedure is presented in Scheme 2. The determination of pyroglutamic acid (secondary amino acid) in hydrolizate was first based on its conversion to the primary amino acid with sodium hypochlorite (NaOCl 2 mM in 0.2 M phosphate buffer, pH = 7.75) [40,41]. An addition of 2,2′ thiodiethanol (3.9 mM in 0.05 M borate buffer, pH = 10.5) was then necessary to limit the excess of sodium hypochlorite which could oxidize OPA or convert primary amines to chloramines and then greatly limit the formation of the fluorescent isoindole [40]. The reaction of the primary amino acid obtained and o-phthalaldehyde and N-acetyl-cysteine (OPA 3 mM/NAC 6 mM in 0.05 M borate buffer, pH = 10.5) forms a fluorescent isoindole compound [40] which can be detected by fluorescence. Potential interferences due to other primary amino acids or primary amines present in the sample were first eliminated by the addition of OPA (30 mM in ethanol/0.2 M phosphate buffered solution (20/80 *v*/*v*, pH = 7.75).

### 3.3. Determination of Tropomyosin

#### 3.3.1. Instrumentation and Software

**Figure 5:**
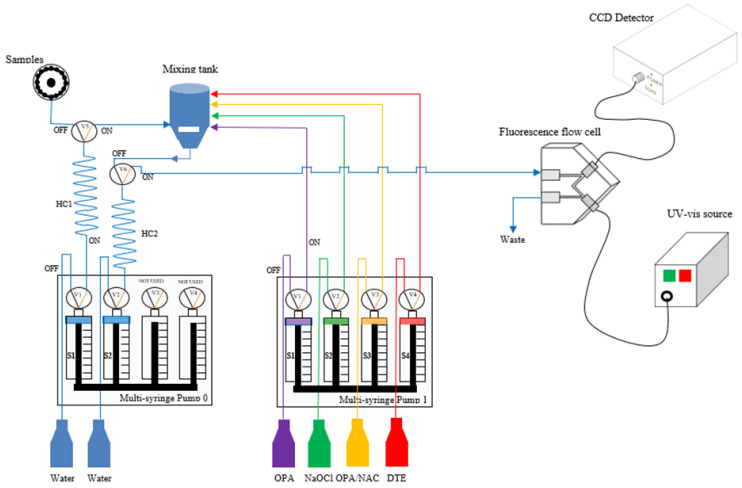
Schematic representation of the MSFIA manifold used for tropomyosin determination (V1–V5 = solenoid valves; S1–S4: syringes; CCD detector: charge coupled device detector).

The experimental set-up of the MSFIA system is described in Figure 5. The propulsion unit of the flow system consisted of two multisyringe burettes (BU-4-S, Crison Instruments, Atella, Spain). Multisyringe pump 0 was equipped with a 5 mL syringe in S1 position and a 2.5 mL syringe in S2 position (Hamilton, manufacturer, city, Switzerland), both of them for ultrapure water dispensing. Four syringes were used in multisyringe pump 1: S1 (1 mL; OPA solution), S2 (2.5 mL; NaOCl solution), S3 (2.5 mL; OPA-NAC reagent), and S4 (2.5 mL; DTE solution). V1 to V5 were standard 3-way solenoid valves. The manifold was designed with poly(tetrafluoethylene) tubing (PTFE; 0.8 mm inner diameter). The derivatization procedure was performed in a 2.5 mL lab-made polymethyl methacrylate (PMMA) mixing tank (1.4 cm i.d. × 2.2 cm height) in which a small magnetic stirrer coated with PTFE (0.9 cm) was used to homogenize the reaction mixture. The detection system was composed of an ultraviolet–visible light source (DH2000, Ocean Optics, Dunedin, FL, USA), a lab-made PMMA flow cell for fluorescence detection with quartz optical windows (0.5 cm optical pathlength in both excitation and emission), and USB2000 miniature spectrometer (Ocean Optics) connected to the computer via a USB interface. Optical fibers of 200 µm and 1000 µm core diameter (FC-UV-200-2 or QP1000-2-UV-VIS Ocean Optics) were used for excitation and emission, respectively. Multysyringe pumps and the detector were controlled by the Autoanalysis 5.0 software package (Sciware Systems SL, Palma de Mallorca, Spain).

#### 3.3.2. Analytical Procedure

The operational steps of the MSFIA system for determination of tropomyosin are detailed in Table 1 and summarized as follows:

Quenching step: 1 mL of sample is picked up into the holding coil HC1 through V5 and injected into the mixing tank using syringe S1 of multisyringe pump 0 then 300 µL of OPA solution are dispensed into the mixing tank by syringe S1 of multisyringe pump 1. The mixture is stirred for 4 min, and during this step OPA reacts with the primary amino acids and blocks their reactivity.

Hydrolysis step: 500 µL NaOCl are added to the mixture in the mixing tank (syringe S2 of multisyringe pump 1) to promote the basic hydrolysis of pyroglutamic acid (secondary amino acid) into a primary amino acid. The mixture is stirred for 2 min.

Derivatization step: in this third step, 500 µL DTE are injected into the mixing tank (syringe S4 of multisyringe pump 1) and the reaction mixture is stirred for 4 min. This addition of DTE removes excess NaOCl. Then, 250 µL OPA-NAC reagent are dispensed into the mixing cell through syringe S3 of multisyringe pump 1. The reaction of OPA with the primary amino acid in the presence of a thiol (NAC) forms a fluorescent isoindole. 

Detection step: 2 mL of the reaction mixture are picked up into the holding coil HC2 through V6 and propulsed toward fluorescence flow cell using syringe S2 of multisynringe pump 0 for detection at λ_ex_ = 335 nm and λ_em_ = 455 nm.

### 3.4. LC-MS/MS Analysis

UHPLC-q-ToF-MS analysis was performed using a UPLC system (Acquity, Waters, MA, USA) interfaced with a quadrupole/time-of-flight mass spectrometer (Q/ToF-MS) equipped with an electrospray ion (ESI) source (Synapt G2 HDM, Waters, MA, USA). Data acquisition and mass spectra treatments were provided by the MassLynx™ software (v.4.1, Waters). 

Optimal separation was achieved on a CORTECS UPLC C18 column (90 Å, 1.6 µm, 2.1 mm × 100 mm) at 40 °C. The mobile phase consisted in A: 0.1% formic acid in water (*v*/*v*) and B: acetonitrile. Elution was performed at a flow rate of 0.4 mL·min^−1^, with a gradient starting at 5% of B and increasing to 95% within 6 min and held for 1 min. The injection volume was 2 µL.

The ESI source contained two individual orthogonal sprays. One spray was for the column eluent while the other was for the internal standard (lock-mass). During each chromatographic run, leucine enkephalin (2 mg L^−1^, C28H37N5O7, MW 555.27, Waters Q-ToF product) was used for lock-mass correction to obtain accurate masses for each organic component eluting from the column. A solution of sodium formate (HCOONa, Waters Q-ToF product) was infused daily in the ESI source to calibrate the instrument. Optimum ESI conditions were found using a 1.0 kV capillary voltage, 20 V and 4 V for sample and extraction cone voltage, respectively, 450 °C desolvation temperature, 120 °C source temperature, 10 L h^−1^ cone gas flow rate, and 900 L h^−1^ desolvation gas flow rate. The ESI source was optimized directly with the samples. Data were collected from 50 to 800 Da in the positive and negative ionization modes. The mass spectrometer was used in its resolution mode. Compounds responding in negative mode were detected as their deprotonated molecules ([M−H]^−^) in the negative mode. MSe fragmentation was performed to confirm the structure of the products. MSe experiments were carried out with a trap collision energy ramp from 5 to 20 eV.

### 3.5. SDS-PAGE Analysis

Sodium dodecyl sulfate-polyacrylamide gel electrophoresis (SDS-PAGE) was performed to visualize the total protein content in the extracts, but also as a purification step before LC/MS analysis. Twelve micrograms of protein extract were briefly heated in Laemmli buffer [42] with dithiothreitol and loaded onto a 15% bisacrylamide gel. Electrophoretic separation was performed at 170 V by use of a Mini-Protean Tetra Cell electrophoresis system (BioRad, Hercules, CA, USA). The separated proteins were visualized by staining with Coomassie brilliant blue G250.

Tropomyosin was excised from the SDS-PAGE gel for mass spectrometric analysis. The SDS-PAGE bands between 34 and 40 kDa (Appendix A) were destained and digested with trypsin, according to the method designed by Abel Rahman et al. [26], before injection into the LC/MS system. Tropomyosin quantification was based on IVELEEELR (sequence) peptide determination, as suggested by Wang et al. [37].

## 4. Conclusions

Samples such as seafood necessarily require a heavy and complex preparation, but automating detection steps can help save time and money. This study has demonstrated that the detection of food allergens in seafood can be achieved indirectly by an MSFIA system via chemical derivatization by OPA/NAC reagent of a secondary amino acid produced by the acid hydrolysis of tropomyosin, the main allergenic protein in shrimp or crab. The analytical protocol developed showed an increase in fluorescence intensity with increasing mass of the prepared shrimp sample, indicating that the method is robust and without matrix effects.

## Figures and Tables

**Table 1:** Detailed multisyringe flow injection analysis procedure for the determination of tropomyosin in shrimp samples.

Step	Multisyringe Pump 0				Multisyringe Pump 1				Mode	Comments
	V1	V2	V5	V6	V1	V2	V3	V4		
1	Ocean optics: get dark current									
2	Ocean optics: get blank current									
3	OFF	ON	OFF	ON	OFF	OFF	OFF	OFF	Dispense	System and tube washing
4	ON	OFF	OFF	OFF	OFF	OFF	OFF	OFF	Pick up	Sample (1 mL) and OPA (300 µL) introduction in mixing tank: quenching of primary amines
5	ON	OFF	ON	OFF	ON	OFF	OFF	OFF	Dispense	
6	Wait 240 s									
7	OFF	OFF	OFF	OFF	OFF	ON	OFF	OFF	Dispense	NaOCl (500 µL) introduction in mixing tank: conversion of pyroglutamic acid into primary amine
	Wait 120 s									
	OFF	OFF	OFF	OFF	OFF	OFF	OFF	ON	Dispense	DTE (500 µL) introduction in mixing tank: excess NaOCl quenching
8	Wait 240 s									
9	OFF	OFF	OFF	OFF	OFF	OFF	ON	OFF	Dispense	OPA-NAC reagent (250 µL) introduction in mixing tank: derivatization
10	Wait 240 s									
13	OFF	ON	OFF	OFF	OFF	OFF	OFF	OFF	Pick up	Reaction mixture aspiration
14	Ocean optics: start measurement									
15	ON	ON	ON	ON	OFF	OFF	OFF	OFF	Dispense	Mixture dispense towards detector and washing of mixing cell
16	Ocean optics: stop measurement									
17	OFF	ON	OFF	ON	OFF	OFF	OFF	OFF	Pick up	Draining mixing cell
18	End of analytical procedure: return to step 3									

**Figure A1:**
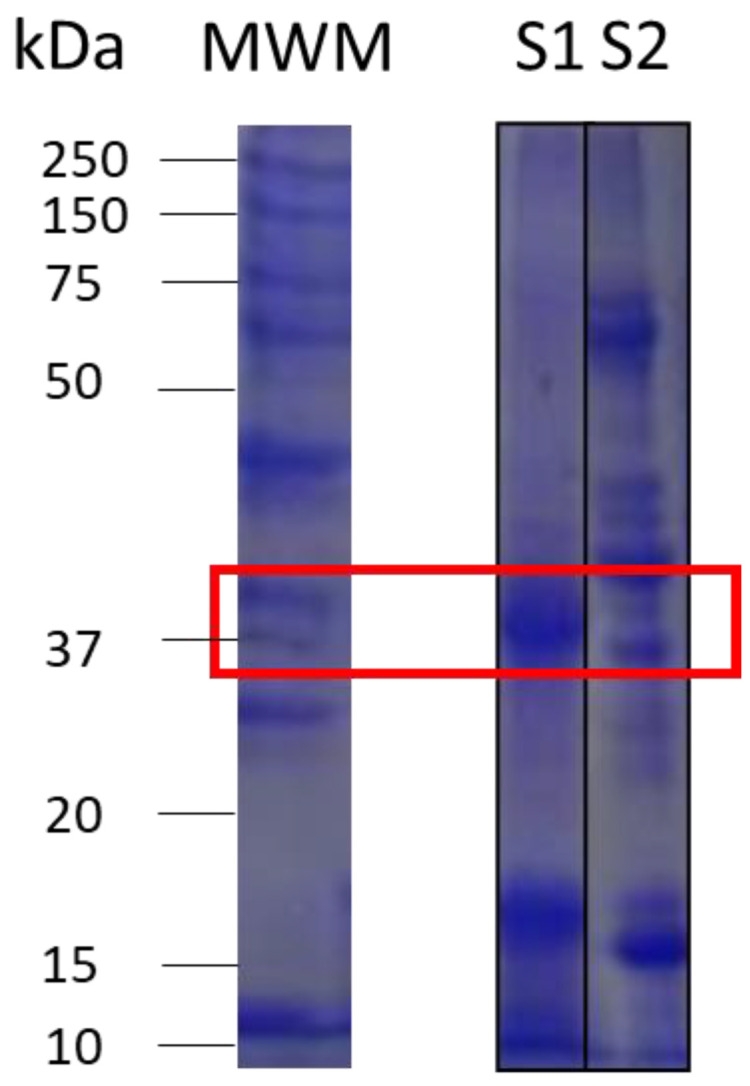
SDS-PAGE of protein extracts of shrimp samples (MWM: molecular weight marker; S1 and S2: shrimp samples 1 and 2).

## Data Availability

No new data were created or analyzed in this study. Data sharing is not applicable to this article.

## Sample Availability

Samples of the compounds are not available from the authors.
